# Intérêt de la plaque en crochet dans les luxations récentes acromio claviculaire stade III et V de Rockwood: à propos de 12 cas

**DOI:** 10.11604/pamj.2014.17.203.2779

**Published:** 2014-03-14

**Authors:** Mohammed Shimi, Mohamed Elidrissi, Atif Mechchat, Abedelhalim Elibrahimi, Abedelmajid Elmrini

**Affiliations:** 1 Service de Chirurgie Ostéoarticulaire B4, CHU Hassan II, Fès, Maroc

**Keywords:** luxation acromio-claviculaire, chirurgie, plaque en crochet, acromioclavicular dislocation, surgery, hook plate

## Abstract

Les luxations acromio-claviculaires récentes stade III et V de Rockwood posent un problème d'indication thérapeutique, un grand nombre d'auteurs s'accordent sur l'intérêt du traitement chirurgical à fin de garantir la stabilité des résultats. Le but de ce travail est d’évaluer les résultats du traitement chirurgical par plaque en crochet. Il s'agit d'une étude rétrospective étalée sur deux ans et demi, comportant 12 patients, tous de sexe masculin présentant une luxation aigue stade III dans 09 cas et un stade V dans 03 cas, l’étiologie été dominée par les AVP dans 75% des cas. Tous les patients ont été opérés par plaque en crochet sans aucune réparation ligamentaire. Après un recul moyen de 17 mois (06 et 30 mois), on a eu un score de Constant moyen de 92,4 (88 et 100), on eu un cas d'ostéolyse au niveau de l'acromion et un cas d'inconfort sous acromial. A travers notre travail et la littérature on peut dire que la plaque en crochet est un moyen simple, peu invasif qui donne des résultats satisfaisants dans la prise en charge des luxations acromio-claviculaires stade III et V avec peu de complications.

## Introduction

**Figure 1:**
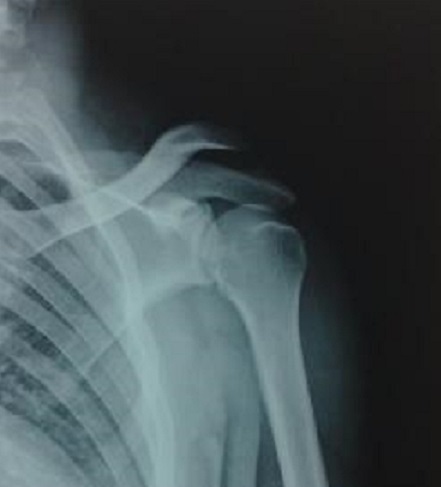
Luxation acromio-claviculaire gauche stade V de Rockwood

Les luxations acromio-claviculaires sont des lésions fréquentes lors des traumatismes directs du moignon de l’épaule, les stades III et V de Rockwood correspondent aux déplacements importants où l′ensemble de la scapula s′effondre, avec le membre supérieur, du fait de la lésion des ligaments trapézoïde et conoïde alors que la clavicule reste en fait dans sa position anatomique (Figure 1). Ces lésions représentent un des sujets les plus controversés en pathologie traumatique ostéo-ligamentaire, tant au niveau du diagnostic lésionnel, qu'au niveau des indications thérapeutiques entre les partisans du traitement conservateur et ceux qui optent pour un traitement chirurgical. Les modalités chirurgicales sont multiples, l'objectif de ce travail est d’évaluer les résultats du traitement chirurgical des luxations acromio-claviculaires stade III et V de Rockwood par plaque en crochet.

## Méthodes

**La série:** Nous avons retenu uniquement les patients présentant une disjonction acromioclaviculaire récente stade III et V, pris en charge dans les 3 premières semaines qui suivent le traumatisme et opérés par plaque en crochet. Les patients vus au delà de ce délai ont été exclus du fait des possibilités différentes concernant la cicatrisation et l’évolution.

Entre janvier 2010 et juin 2012, on a pu retenir 12 patients, tous de sexe masculin, L′âge moyen était de 38ans avec des âges extrêmes entre 22 et 55 ans. 7 de nos patients étaient des travailleurs manuels, 4 sans profession, et 1 sportif professionnel. Le mécanisme fut direct dans 80% des cas, les étiologies ont été dominées par les AVP dans 08 cas et les accidents de sport dans 03 cas et dans 1 cas une chute sur l’épaule. Tous nos patients avaient bénéficié d′une radiographie de l′épaule de face qui a révélé une luxation acromio-claviculaire stade III de Rockwood dans 09 cas et un stade V dans 03 cas.

**Figure 2:**
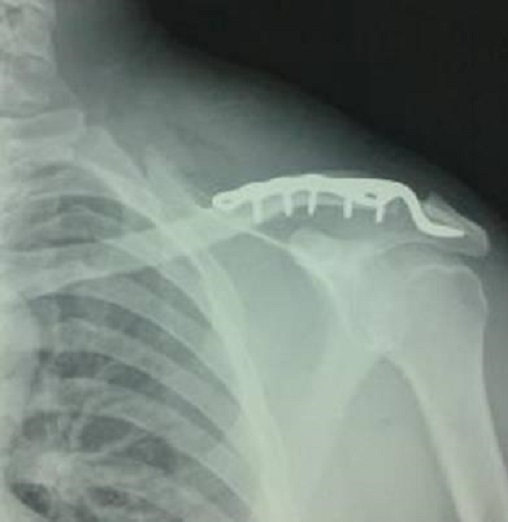
Réduction et stabilisation par plaque en crochet

**La technique opératoire:** Tous nos patients ont été opérés sous anesthésie générale; l′installation du patient se fait sur une table ordinaire en décubitus dorsal et en position demi assise; le bilan lésionnel évalue l′état de la chape trapézo-deltoidienne, l′état de surfaces articulaires, du pivot coraco-claviculaire qui a été toujours rompu; la réduction est faite par plaque dont le crochet est introduit sous l'acromion sans dissection (Figure 2).

Aucune réparation ou plastie ligamentaire n'a été faite. La rééducation se fait après environ une semaine, dès que la douleur diminue, ce qui contribue à préserver une gamme normale de mouvements et du tonus musculaire, la levée du poids est commencée progressivement à partir de 45 jours. L'ablation de la plaque a été faite à partir du 3ième mois (3 et 8 mois) chez 08 patients, alors 4 patients n'ont pas voulus enlever la plaque.

### Les méthodes d’évaluation

Clinique: utilisant le score de Constant. - Radiologique: sur des radiographies de l’épaule de face centrés sur l'articulation acromio-claviculaire on apprécie la qualité de la réduction, les calcifications et la lyse osseuse sur l'acromion.

## Résultats

**Figure 3:**
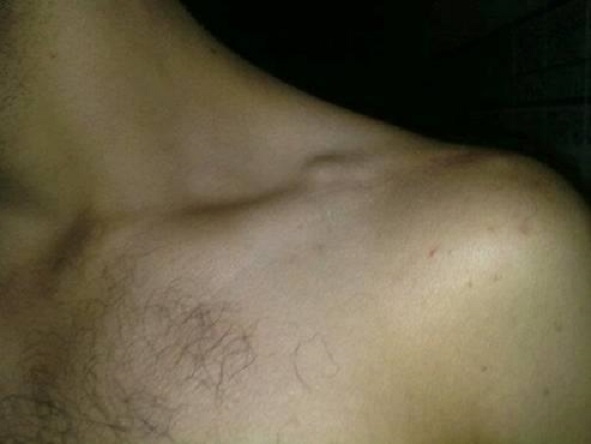
Aspect clinique à 24 mois de recul

**Figure 4:**
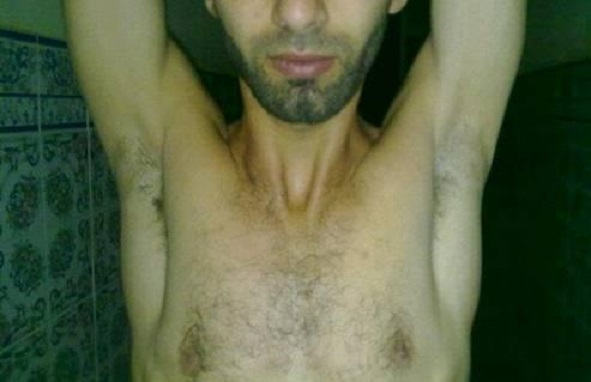
Résultat fonctionnel à 24 mois de recul plaque toujours en place

Le score de Constant a été mesuré complètement (sur 100 points) chez les 12 patients revus cliniquement il a été en moyenne à 92,4 (88 et 100). Il est statistiquement plus faible du côté atteint, il est néanmoins excellent ou bon chez tous les malades tous les patients ont pus reprendre leurs activités professionnelles au bout de 03 mois en moyenne avec des extrêmes entre 02 et 09 mois (Tableau 1, Figure 3, Figure 4).

**Tableau 1:** Récapitulatifs des patients opérés avec résultats fonctionnels

Cas	Age	Profession	Etiologie	Stade	Score De Constant	Résultat	Complications
01	22	Étudiant	A.sport	III	100	Excellent	Aucune
02	31	Commercant	AVP	III	95	Excellent	Aucune
03	45	Maçon	AVP	V	88	Bon	Aucune
04	24	Sans	AVP	III	90	Excellent	Aucune
05	32	Ouvrier	A.sport	V	88	Bon	Ostéolyse
06	38	Mécanicien	AVP	III	100	Excellent	Aucune
07	55	Agriculteur	AVP	III	85	Bon	Géne sous acromial
08	27	Sportif	A.sport	III	100	Excellent	Aucune
09	30	Sans	chute	III	90	Excellent	Aucune
10	24	Ouvrier	AVP	V	88	Bon	Aucune
11	22	Agriculteur	AVP	III	95	Excellent	Aucune
12	27	Bureau	AVP	III	90	Excellent	Aucune
Moyenne					92,4		

Les radiographies postopératoires et les radiographies de contrôle ont montrés une bonne réduction de la luxation, l'absence de calcifications, la perte de réduction.

### Les complications

On n'a pas noté de complications particulières en peropératoire. Aucun cas d'infections n'a été noté. Un cas d'ostéolyse au niveau de l'acromion provoquée par le crochet, a été observé et a motivé l'ablation de la plaque au 6ième mois. Un patient a rapporté une gêne au moment de la mobilisation de l’épaule en abduction par malposition de la plaque chez qui on a réalisé l'ablation au 3ième mois.

## Discussion

Les luxations acromio-claviculaires stade I et II de Rockwood relèvent d'un traitement orthopédique, les stades IV et VI sont chirurgicales du fait du grand risque d'instabilité [1], le problème se pose surtout pour Les stades III et V de Rockwood qui restent sujets de controverse, certains auteurs préconisent le traitement orthopédique en considérant qu'il donne des résultats égaux voir meilleurs que le traitement chirurgical avec moins de complications et un retour plus rapides aux activités antérieures [2, 3]. Cependant chez des travailleurs de force et les sportifs de haut niveau il faut restaurer une anatomie normale en assurant une stabilité et une bonne congruence articulaire d'où l'intérêt d'un traitement chirurgical pour ce type de lésion [4–8].

Johan Von Heideken [9] considère que la cicatrisation des ligaments coraco-claviculaires est nécessaire pour avoir un bon résultat et éviter la dégradation de la fonction par instabilité ou reluxation.

Plusieurs techniques chirurgicales ont été proposée pour le traitement de ces luxations acromio-claviculaires tels que l'embrochage acromio-claviculaire, le vissage coraco-claviculaire, les ligamentoplasties de Weaver-Dunn, les ligaments synthétiques ou autres, les différentes publications montrent que les résultats sont similaires mais elles diffèrent dans les complications [10, 11] Récemment des résultats satisfaisants ont été rapportés par l'arthroscopie mais elle reste très exigeante sur le plan technique et nécessite une courbe d'apprentissage [12].

La fixation par plaque en crochet est une technique qui a été introduite dans la prise en charge des luxation acromio-claviculaires, c'est une technique peu invasive, reproductible, l'objectif étant de maintenir la réduction en assurant une stabilité dans le plan vertical et horizontal en attendant la cicatrisation ligamentaire qui va permettre de maintenir la réduction même après ablation de la plaque. La réparation ligamentaire dans les luxations fraîches n'est pas obligatoire, Alexander Di Francesco et al [13] ont réalisé une étude des ligaments par IRM après traitement par plaque en crochet chez 42 patients porteurs de luxation acromio-claviculaire stade III et V, et ont montré une cicatrisation des ligaments coraco-claviculaire chez 37 patients soit 88% des cas. Nos résultats sont similaires à ceux des différentes séries retrouvés dans la littérature et adoptant la même technique chirurgicale [6, 14, 15] avec un score de constant entre 88 et 94 selon les séries.

L'ablation de la plaque est souvent réalisée après 3 mois, mais une étude biomécanique sur cadavres a montrée que la plaque en crochet permet des mouvements physiologiques de la clavicule, ainsi il est possible de garder la plaque au delà de la cicatrisation ligamentaire chez des sujet asymptomatique comme chez nos 04 patients chez qui on n'a pas enlevé la plaque. Les complications sont minimes essentiellement l'inconfort sous acromial et la lyse au niveau de l'acromion par le crochet de la plaque, la gène esthétique [6, 14–16] et les risques liés à une intervention pour l'ablation de la plaque [6, 16]. La possibilité de reluxation existe après ablation de la plaque de ce fait Koukakis et al. [11] considèrent que la dissection minutieuse en respectant l'insertion du trapèze et du muscle deltoïde sur la clavicule empêcherait la reluxation après ablation de la plaque.

## Conclusion

Malgré un recul relativement court, et le petit nombre de cette série on peut conclure que la plaque en crochet est une technique simple, peu invasive, reproductible pour les luxations acromio-claviculaires stade III et V de Rockwood avec un faible taux de complications.
